# Influence of Dispersed Reinforcement on Mechanical Properties of Stabilized Soil

**DOI:** 10.3390/ma14205982

**Published:** 2021-10-11

**Authors:** Maciej Miturski, Andrzej Głuchowski, Wojciech Sas

**Affiliations:** 1 Department of Geotechnical Engineering, Institute of Civil Engineering, Warsaw University of Life Sciences—SGGW, 02-787 Warsaw, Poland; 2 Water Centre, Warsaw University of Life Sciences—SGGW, 02-787 Warsaw, Poland; andrzej_gluchowski@sggw.edu.pl (A.G.); wojciech_sas@sggw.edu.pl (W.S.)

**Keywords:** stabilization, subbase, soil reinforcement, elastic, tangent module, secant module, unconfined compressive strength test

## Abstract

Stabilized soils are commonly used as part of pavement construction in highway engineering. The everyday use of this material makes it necessary to classify it. One of the basic methods of determining the mechanical properties of a material is the unconfined compressive strength (UCS) test, from which the material elasticity can be determined. The scope of the research included the design and making of soil mixtures stabilized with polypropylene fibers modified cement. This paper presents the effect of the amount of dispersed reinforcement on the maximum compressive strength, the secant modulus at half the ultimate stress ($E_{50}$), the secant modulus at the ultimate stress ($E_{s}$), and the tangent modulus ($E_{t}$). The materials chapter characterizes the soil, cement, and dispersed reinforcement used. The test methods section describes the tests performed and the procedure for interpreting the results. The results section describes the relationship between elastic modulus and compressive strength. The discussion section compares the obtained results with the works of other authors. The work is concluded with a summary containing the most important conclusions resulting from the work.

## 1. Introduction

Soil improvement can be divided into two basic techniques: stabilization and reinforcement. In general, soil stabilization improves soil properties by blending and mixing other materials [1]. The combination of soil and binder results in a composite material. The idea of stabilization itself has been known for 5000 years [2]. At present, this technique is mainly used in the construction of linear structures and deep soil improvement [3]. In road engineering, it is primarily used as a subbase or top layer for local road construction. Due to the large volumes of soil masses used in highway engineering, the use of improvement techniques has a reasonable explanation [4]. Another commonly used material for highway construction improvement is Recycled Concrete Aggregate (RCA) [5]. The stabilization process can be divided into three primary groups: traditional stabilizers (hydrated lime, Portland cement, and fly ash), byproduct stabilizers (cement kiln dust, lime kiln dust, and other forms of byproduct lime), and nontraditional stabilizers (sulfonated oils, potassium compounds, ammonium chloride, enzymes, and polymers) [6]. Of all these stabilizers, cement is widely used to improve the soil’s strength properties [7,8,9,10,11,12].

The second improvement technique is soil reinforcement. This method has been known since ancient times. Moreover, it occurs naturally in the form of the root system of plants [13]. In the 20th century, Vidal [14] was the first to present the concepts and principles of soil reinforcement. He showed that the implementation of reinforcing elements into the soil mass increases the shear resistance of the medium [14,15]. Currently, in addition to the commonly used geosynthetics [16,17], short polypropylene fibers are also used for soil reinforcement. This technique is seen as relatively new in geotechnical engineering. The application of fiber reinforcement aims to capture and redistribute the load due to their tensile strength, mobilize a wider soil mass, and then improve the mechanical response of the material [13]. Fiber reinforcement has been successfully applied in highway engineering in cement base layers [18,19,20,21]. Both of these techniques have some disadvantages. Stabilized soils have low tensile strength and reinforced soils have a significantly lower stiffness than stabilized soils. Combining these two techniques in the form of a reinforcement added to the stabilized soil allows a significant improvement of the mechanical properties [22,23,24,25,26].

For years, many studies have been conducted on the effect of dispersed reinforcement on the mechanical properties of soils and stabilized soils. Most often, polypropylene fibers (PP) are used as reinforcement elements [8,27,28,29,30]. The primary effect of reinforcement is to increase strength during unconfined compression (UC) and has been studied by many researchers [23,24,30,31,32,33,34]. Alongside polypropylene fibers, other fibers, including natural fibers, are also used. Research on the application of such reinforcement is often performed by investigators [12,22,26,35]. Tests conducted under triaxial compression of stabilized soil with the addition of reinforcement showed that the use of additives in the form of reinforcement increases the angle of internal friction and cohesion of such material.

Furthermore, it was observed that the significance of the reinforcement content decreases with an increase in confining pressure [36]. Despite the increase in strength, a decrease in elastic modulus was observed by using dispersed reinforcement. To predict the effect of the fibers on the soil or stabilized soil, the reinforcing elements’ distribution must also be taken into account. This can have a significant influence on the results [37]. Due to the possibility of using the material in subbases, the increase in tensile strength is also an essential factor. Two basic types of tests can be distinguished to determine tensile strength. The first method is to determine the tensile strength directly. The second is an indirect method, also known as the Brazilian test [38]. Studies conducted by a number of investigators have shown significant increases in tensile strength (ITS) by using dispersed reinforcement in stabilized soils [12,20,32,39,40,41]. Researchers’ efforts are not only limited to studying the effect of reinforcement on strength parameters but also to predict the impact of fiber addition. The mechanism of action of dispersed reinforcement in soil has been described by Gray and Ohashi, among others [42,43]. To predict the effect of applied reinforcement, many researchers attempt to create a model and derive an equation that could predict changes in properties, such as compressive strength, tensile strength, strain, stiffness, modulus values, and other parameters [12,25,40,44,45]. A relationship between mixture porosity and unconfined compressive strength was also observed. They reduced the compacted mix’s porosity, significantly improving the strength of both soils stabilized with and without fiber addition. The compressive strength was shown to increase non-linearly with decreasing porosity of the compacted mix [20,24,46]. The dispersed reinforcement does not only influence the mechanical properties of the hardened mixes. It also influences the physical properties, such as optimum mixture moisture and the mix’s maximum dry density. Adding fibers to the stabilized soil increases the optimum moisture content and decreases the maximum dry density [12,47].

To fully understand the effect of distributed reinforcement on stabilized soils, it is also necessary to study such reinforcement’s impact on the deformation characteristics. To determine the correct modulus values from unconfined compressive strength (UCS) tests without using extensometers mounted directly on the specimen. It is necessary to analyze the measurement data obtained and interpret the data correctly. This is due to the first stage of the test’s inaccuracy when the piston is adapting to the specimen. This paper discusses the effect of dispersed reinforcement on the mechanical properties of tested mixtures. The impact of measurement data interpretation on tangent and secant modulus values and the changes that occur in the modulus values by using polypropylene (PP) fibers is discussed. The results presented here allow for a better understanding of the relationship between deformation characteristics and strength characteristics. The main subject of this paper is the influence of dispersed reinforcement on the mechanical and physical properties of soil and binder mixtures. Moreover, the paper discusses the influence of interpretation on the results, which is often overlooked in many works.

## 2. Materials and Methods

For this paper’s purpose, tests were conducted on 9 mixtures consisting of soil, binder, and dispersed reinforcement. The soil was classified as clSa and is described in detail in Section 2.1 CEM III (Górażdże Cement S.A., Chorula, Poland) blast furnace cement was used as a binder; see Section 2.2 for a more detailed description. Polypropylene fibers were used as dispersed reinforcement. A detailed description of the reinforcement used is included in Section 2.3. All mixtures are classified in accordance with European standards. The specimens were compacted at the optimum moisture content and had a slenderness of 1. The complete procedure for specimens and their determination are presented in Section 2.4. All samples were subjected to the care process described in Section 2.5. The unconfined compressive strength tests were performed using Instron apparatus, and the dust test was performed at constant load increase. The apparatus and test conditions are described in detail in Section 2.6. The interpretation method was based on the assumption of a linear stress-strain relationship in the first phase of the test. Section 2.7 describes in detail how to interpret the measurement data obtained from the test apparatus. 

### 2.1. Soil

The soil used for this paper was taken from a depth of 3 m from an urban area. The basic physical and mechanical characteristics of the investigated soil were identified. In order to determine the basic physical characteristics, aerometric analysis was performed using a complete set of sieves. Aerometric analysis was also performed to distinguish the finer fractions. In order to calculate the plasticity index (PI), liquidity limits (w_L_) and plasticity limits (w_P_) were measured. The liquid limit (w_L_) was measured using the Casagrande apparatus, while the plastic limit was determined by the rolling method. 

**Figure 1:**
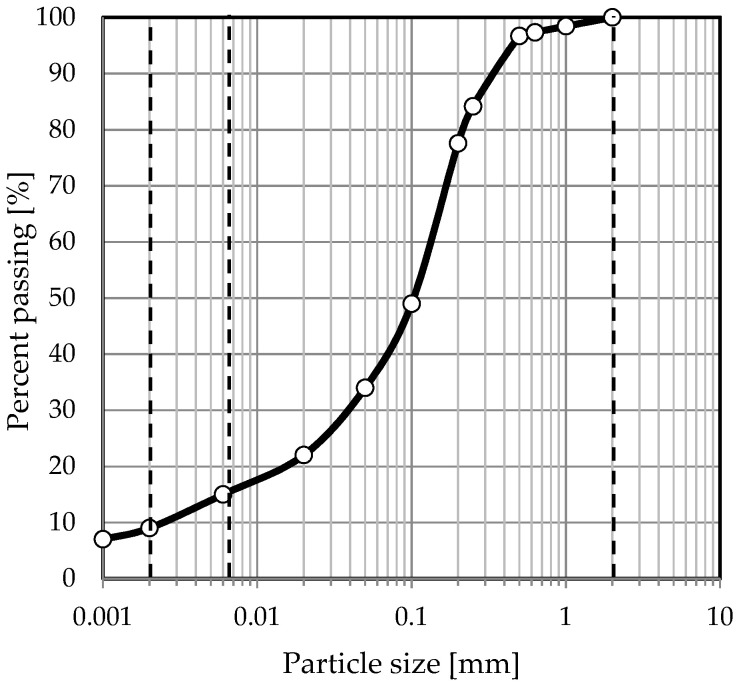
Grain size distribution curve of soil binder.

All determinations were made according to European standards [48]. All the main information is given in Table 1, and the grain size curve of the soil under study is shown in Figure 1. Soil was classified as clayey sand (clSa) [49].

### 2.2. Binder

Blast furnace cement (BFC) was used as the binder. This type of binder is often used as a stabilization material in highway engineering. This choice is often motivated by economic reasons. Appropriate storage conditions were provided to ensure that the cement had adequate properties during stabilization. The binder was stored in a dry room, not exposed to moisture. All samples using the binder were made before the expiration date. Basic information about the binder is provided in Table 2.

### 2.3. Reinforcement

Polypropylene (PP) fibers were used as dispersed reinforcement. All the fibers used were of the same length. Basic information about the reinforcement used is included in Table 3.

### 2.4. Sample Preparation Procedure

Sample preparation always followed the same procedure. First, the soil, which is the basic material, was prepared and decomposed into fine fractions. This process was done to avoid large soil fragments sticking together, preventing proper mixing of the soil with the reinforcement. In the next stage, the components were dosed. First, dispersed reinforcement was added to the weighed quantity of soil and mixed with dry soil. In the case of mixtures without the dispersed reinforcement, the stage of adding fibers was skipped. In the next step, the cement was dosed into a mix of soil and reinforcement. After dosing the cement, another mixing process took place. In the third step, water was added to the mixture of soil, reinforcement, and cement. All components of the mixture were measured by weight.

All the ingredients were thoroughly mixed until a homogeneous mixture was obtained. Stabilized soil samples were made using the prepared mixtures. The samples were made according to the standards [50]. The samples were formed in molds of two parts with a cylindrical shape. The height of the prepared sample was 8 cm and the diameter was equal to 8 cm. All samples were compacted with an energy of 0.59 [J × m^−3^]. Six samples were prepared in one process. Three of them were for testing after 7 days and the rest after 28 days. The care process started according to the standard [51] with the production process’ completion. 

Nine types of mixtures were prepared; each mixture contained a characteristic cement and fiber content. All mixtures were compacted at the optimum moisture content for the mix; therefore, optimum moisture content (OMC) and maximum dry density (MDD) were determined for each combination of soil, cement, and fibers used. 

**Figure 2:**
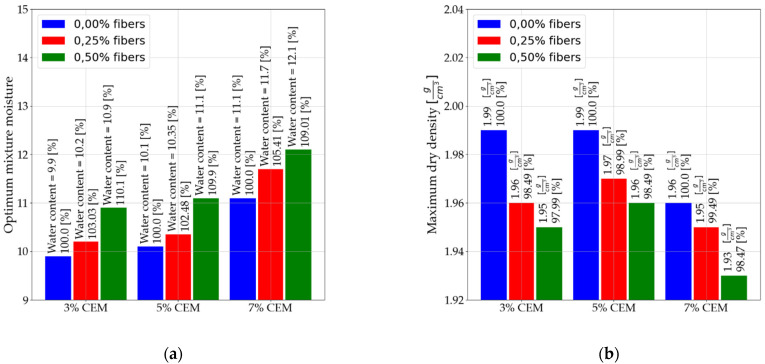
The figure shows (**a**) optimum moisture content of nine mixtures and (**b**) maximum dry density of the nine mixtures.

The specific contents of each mixture component and their identification are shown in Table 4. Figure 2 shows the optimum moisture contents and MDD for each mixture used.

### 2.5. Sample Care Process

The care process for each sample began when the sample was compacted. The care process included two phases. The first phase involved curing in a room with constant humidity and a temperature of 22 °C ± 2 °C. The second phase consisted of submerging the stabilized soil sample in a water tank until testing. Each sample was removed from the mold after 24 h when the initial strength was achieved. This process was designed to avoid damage to the fresh sample. The process was made possible by using a split mold. Samples intended for testing on day 7 were placed in the water tank after 3 days, while samples intended for testing after 28 days were placed in the water tank after 21 days. Each sample was removed from the water and dried out on the testing day. Each sample was then weighed and tested.

### 2.6. Measuring Instruments

The unconfined compressive strength test was performed using a Universal Testing System made by Instron (Norwood, MA, USA), model 5982. The load limit that the press can apply and the record is 100 KN. The measuring machine is equipped with a displacement recorder and a load cell. The instruments provide a force accuracy of ±0.5% and a displacement accuracy of ±0.01 mm. Data is recorded at a frequency of up to 2.5 kHz. All data is stored in Bluehill software (Instron, Norwood, MA, USA). A constant load increment was programmed into the software during the test, which was 16 kN/min. The accuracy of the load speed reference is subject to an inaccuracy that may amount to 0.1% of the load speed reference with the equipment used. Before each test, the instrument was calibrated to avoid measurement errors. Key data are summarized in Table 5.

### 2.7. Interpretation of Measurement Data

**Figure 3:**
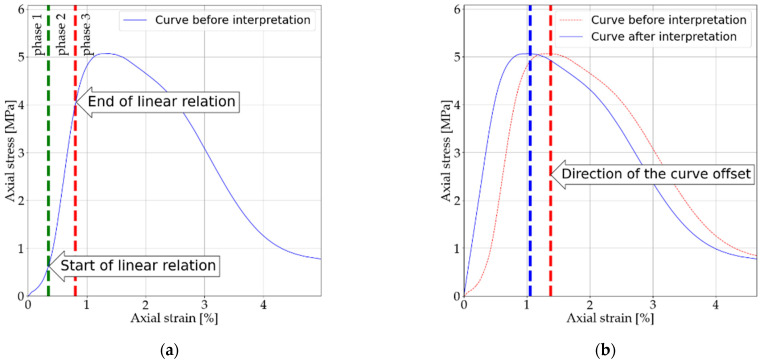
(**a**) The boundary of the start and end of a linear relationship. Division into three study phases (phase 1, phase 2, phase 3). (**b**) Example interpretation of measurement data.

A typical test process can be divided into three phases. The first phase shows a nonlinear stress-strain relationship, and this is the phase in which the piston is adapted. According to the measurement data, there is an elastic increase of the tested material in this phase. The second phase is a linear relationship of stress and strain, and this is the phase in which Hook’s law is applied. The third phase involves the nonlinear behavior of the specimen. The interpretation of the results is to consider the linear nature of the stress-strain curve from the test’s beginning. For this purpose, each test was analyzed to determine the transition point from phase one to phase two. The determination of the characteristic point was based on observing the variation of the slope of the function. When successive steps did not result in significant changes in the slope, it was assumed that a linear relationship had already been established. Once this boundary was determined, an adjustment was made to the measurement data so that the test was linear from the test’s start. The correction also took into consideration the displacement during the measurement. The interpretation performed in this way did not consider the initial modulus, which usually has a higher value than the secant or tangential moduli. The interpretation scheme is illustrated in Figure 3. After interpretation, only two stages could be distinguished. Phase two was the linear relationship between stress and strain, and phase three was the transition from linear to nonlinear.

## 3. Results

**Figure 4:**
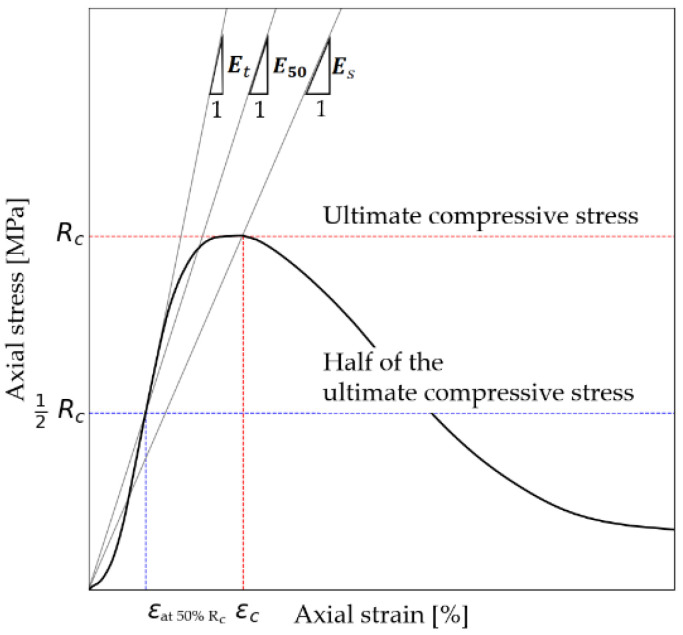
Analyzed elastic modules on the specimen before interpretation.

The results were compiled from data recorded during testing using Bluehill software. The chapter is divided into four subsections. Section 3.1 compares the unconfined compressive strength (UCS) at 7 days and 28 days and classifies each mix into a strength group according to the standard. The unconfined compressive strength according to the standard [51] was denoted as R_c_. Section 3.2 includes the effect of dispersed reinforcement on the modulus values obtained for unconfined compressive strength. The authors analyzed three types of modulus. The first modulus was the secant modulus $E_{s},\;$ determined for the most considerable obtained value of unconfined compressive strength. The second modulus was the modulus determined for half of the ultimate compressive stress and was designated as $E_{50}$. The third modulus considered was the tangent modulus $E_{t}$ to half of the ultimate compressive stress. The modules before interpretation are shown in Figure 4. Section 3.3 presents observations of the results presented in the previous two subsections. Section 3.4 presents the relationships between the obtained values of unconfined compressive strength from Section 3.1 and the obtained values of moduli from Section 3.2.

The interpretation of the measured data did not affect the results in Section 3.1. Section 3.2 analyzes the obtained results resulting from the interpretation of the tests. In Section 3.4, an analysis is made for the final results after the analysis. The results presented include data collected from 81 samples tested.

### 3.1. Influence of Fibers on unconfined compressive strength

**Figure 5:**
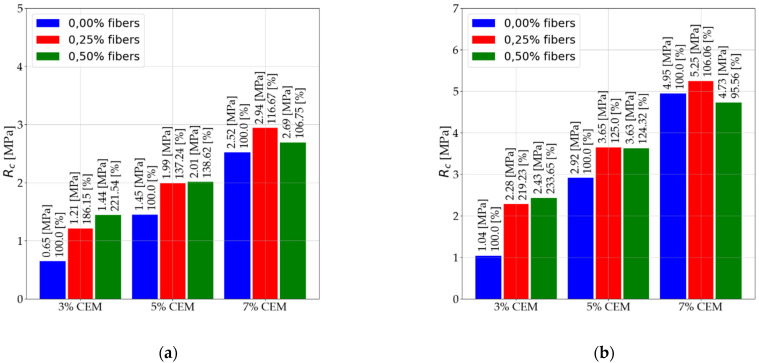
unconfined compressive strength (**a**) after 7 days of care and (**b**) after 28 days of care.

The results presented are for the average unconfined compressive strength for nine mixtures tested after 7 and 28 days of care. Each average result was determined from three tests. A total of 81 samples were tested. The results were summarized in a bar graph and tabulated. The results are presented in Figure 5 and Table 6.

The presented results show that similar results are obtained for the same content of the binder and reinforcement independent of the number of days of treatment.

- The use of dispersed reinforcement in the amount of 2.5‰ and 5‰ in the mixtures with 3% cement content results in an increase of compressive strength up to 133.65%
- The use of dispersed reinforcement in mixtures with a 7% binder may decrease compressive strength.
- The use of 5‰ reinforcement in addition to the early strength for mixes with 3% content does not increase compressive strength; it may even cause a decrease in compressive strength.

Based on the results, it may be assumed that the application of dispersed reinforcement above a certain amount in relation to the dry mass of soil loses its significance because of the lack of increase in the strength of the tested mixtures. However, the use of up to 2.5‰ of fiber brings notable effects. In order to increase the effectiveness of the applied reinforcement, it should be used in mixtures with low binder content. The summary of results and strength classification is presented in Table 6. It is important to note that, for different types of reinforcement, different fiber lengths, mix proportions, and moisture, content could significantly differ.

### 3.2. Influence of Fibers on Modules

**Figure 6:**
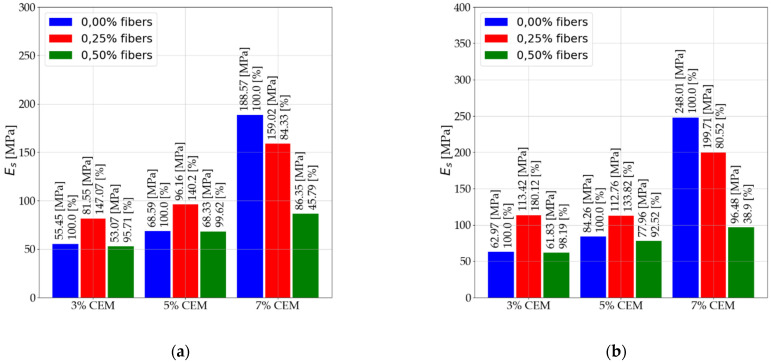
Values of secant modulus $E_{s}$ after 7 days of care (**a**) before interpretation and (**b**) after interpretation.

The next subsection presents information on the effect of dispersed reinforcement on the elastic of stabilized mixtures. The subsection presents results for the three types of elastic moduli analyzed. The results presented are for pre-and post-interpretation data for secant modulus ($E_{s}$) and secant modulus ($E_{50}$). The interpretation does not affect the obtained results of the tangent modulus ($E_{t}$) determined at half-ultimate compressive strength. Results are presented for tests performed after 7 and 28 days. Figure 6 shows the results for the secant modulus ($E_{s}$) after 7 days of care.

**Figure 7:**
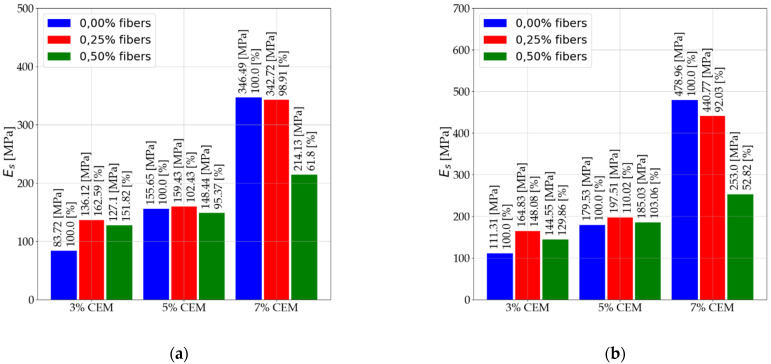
Values of secant modulus $E_{s}$ after 28 days of care (**a**) before interpretation and (**b**) after interpretation.

**Figure 8:**
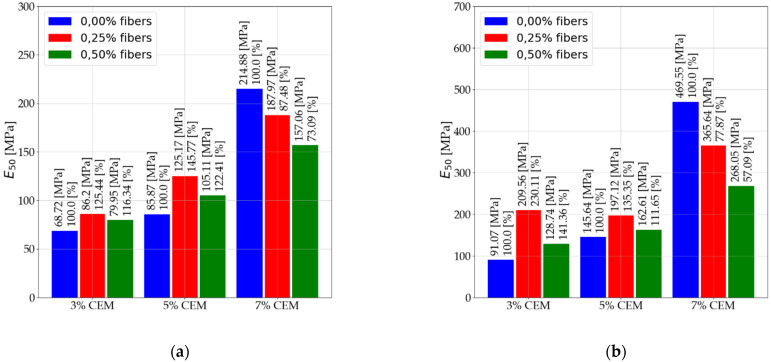
Values of secant modulus $E_{50}$ after 7 days of care (**a**) before interpretation and (**b**) after interpretation.

**Figure 9:**
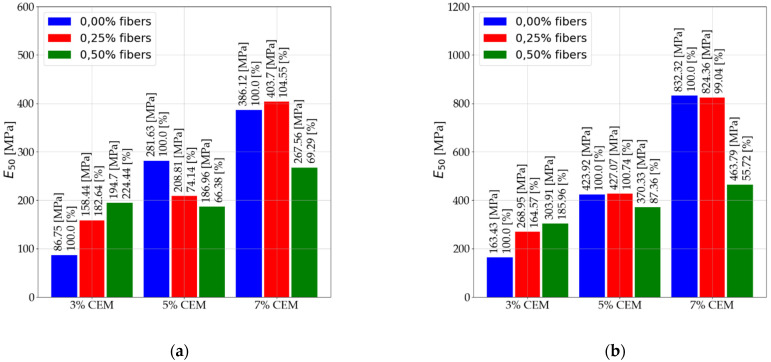
Values of secant modulus $E_{50}$ after 28 days of care (**a**) before interpretation and (**b**) after interpretation.

**Figure 10:**
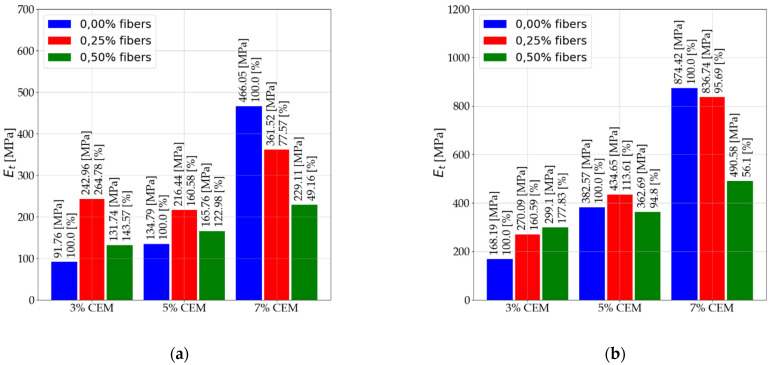
Values of tangent modulus $E_{t}$ (**a**) after 7 days of care and (**b**) after 28 days of care.

Figure 7 shows the results for the secant modulus ($E_{s}$) after 28 days of care. Figure 8 shows the results for secant modulus ($E_{50}$) after 7 days of care. Figure 9 shows the results for secant modulus ($E_{50}$) after 28 days of care. The results presented in Figure 6, Figure 7, Figure 8 and Figure 9 are divided into results before interpretation and after interpreting the experimental data. Figure 10 shows the results for tangent modulus ($E_{t}$) after 7 and 28 days.

### 3.3. Observations

According to the results in Figure 6 and Figure 7, the following were observed:

- An increase in elastic modulus ($E_{s}$) of stabilized soils that contain 3% and 5% binder due to 2.5‰ dispersed reinforcement. Mixtures (C112, C114, C212, C214).
- Elastic modulus reduction ($E_{s}$) of stabilized soils with 5% addition of dispersed reinforcement (C122, C124, C222, C224) in relation to mixtures with 2.5‰ fiber content.
- The use of dispersed reinforcement in soils with 7% (C312, C314, C322, C324) binder addition causes a significant decrease in elastic modulus.
- The modules after interpretation increased on average by 22.71% compared to the baseline values.
  According to the data in Figure 8 and Figure 9, the following were observed:
- An increase in elastic modulus ($E_{50}$) of stabilized soils containing 3% and 5% binder due to 2.5‰ dispersed reinforcement. Mixtures (C112, C114, C212, C214). However, for mixture C214, the increase can be ignored due to its small increase.
- Reduction in elastic modulus ($E_{50}$) of soils stabilized with 5% addition of dispersed reinforcement (C122, C222, C224) in relation to mixtures with 2.5‰ fiber content. Note the mix C124, which recorded an increase in elastic modulus.
- The use of dispersed reinforcement in soils with 7% (C312, C322, C324) binder addition results in a significant elastic decrease. The C314 mix recorded a slight reduction in elastic modulus compared to the C304 mix.
- The post-interpretation modulus increased compared to the baseline values by an average of 81.43%.
  According to the data in Figure 10, the following were observed:
- Increase in elastic modulus ($E_{t}$) of stabilized soils containing 3% and 5% binder as a result of dispersed reinforcement for mixtures (C112, C114, C124, C212, C214).
- Elastic reduction ($E_{t}$) of stabilized soils with 5% addition of dispersed reinforcement (C122, C222, C224) in reference to mixtures with 2.5‰ fiber content. Note the C124 mixture, which recorded an increase in elastic modulus.
- The use of dispersed reinforcement in soils with 7% (C312, C322, C324) binder addition results in a sharp decrease in elastic modulus. The C314 mixture recorded a slight reduction in elastic compared to the C304 mix.

Interpretation of the results did not affect the value of the tangent modulus ($E_{t}$), and after interpretation, values comparable to the secant modulus ($E_{50}$) were obtained.

Table 7 and Table 8 present the results showing the effect of interpretation on the value of ($E_{s}$) and ($E_{50}$) modules.

### 3.4. Relationship of Soil Stabilized Elastic to Compressive Strength

**Figure 11:**
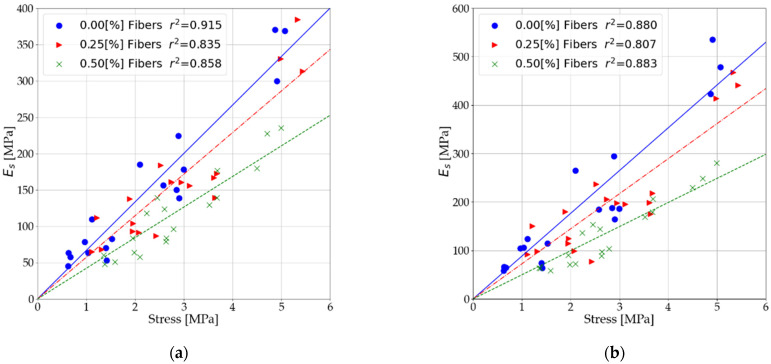
Influence of fibers on the relationship of secant moduli and ultimate compressive strength (**a**) before interpretation of measurement data and (**b**) after interpretation of measurement data.

**Figure 12:**
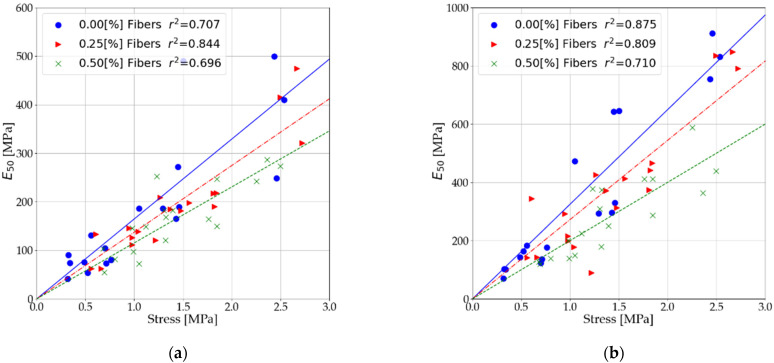
Influence of fibers on the relationship of secant moduli at 0.5 R_c_ and ultimate compressive strength (**a**) before interpretation of measurement data and (**b**) after interpretation of measurement data.

**Figure 13:**
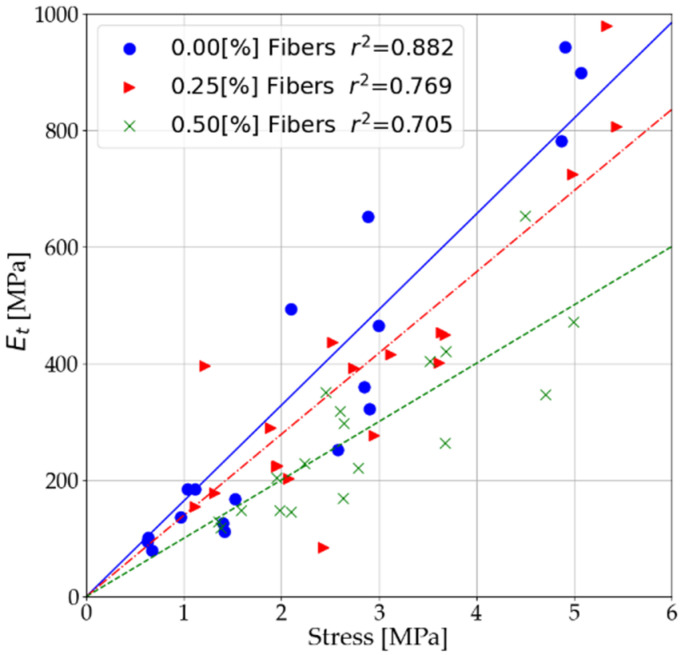
Influence of fibers on the relationship of tangent moduli at 0.5 R_c_ and ultimate compressive strength.

Based on the results presented in Section 3.1 and Section 3.2, the relationships between the modulus and unconfined compressive strength values were verified. The relationships were presented both before and after interpretation of the measurement data. The statements include both tests performed after 7 and after 28 days. Figure 11 shows the relationship between secant modulus ($E_{s}$) and unconfined compressive strength. Figure 12 presents the relationship between the secant modulus ($E_{50}$) and unconfined compressive strength. Figure 13 shows the relationship of shear modulus ($E_{s}$) and unconfined compressive strength.

As a result of dispersed reinforcement in stabilized soils, the relationship of elastic modulus and unconfined compressive strength is modified. This modification consists of the weakening of the modulus values in relation to the unconfined compressive strength. Two main reasons for this response can be distinguished:

- With dispersed reinforcement, the increase in unconfined compressive strength is higher than the increase in elastic of soils stabilized at low binder content.
- The use of dispersed reinforcement in mixes with 7% binder content causes a significantly more significant decrease in the stabilized soil’s elastic than its strength.

Using a simple relationship, it is possible to approximate the values of the elastic modulus for cement-stabilized soils, taking into account different contents of dispersed reinforcement.

(1) $$E_{\left(s,\;50,t\right)}=A\cdot R_{c}$$

where $R_{c}$ is the unconfined compression strength (UCS) and *A* is the slope of the trend line, the parameter is given in Table 9.

## 4. Discussion and Conclusions

We compared the presented research results with works published by other authors [10,12,23,31,52]. Note that the comparable changes in physical and mechanical properties are due to the application of dispersed reinforcement to stabilized soils. The use of distributed reinforcement in the form of polypropylene fibers and natural fibers leads to a decrease in the volume density of mixtures and an increase in optimum moisture content. The results obtained are consistent with the results presented by other researchers [12,23]. In the publication [23], Ayeldeen and Kitazume noted that using polypropylene fibers above 5*‰* causes a decrease in the tested specimens’ unconfined compressive strength. The higher the binder content, the greater the decreases. The research presented in this paper also noted the reduction in unconfined compressive strength. According to the study, the strength reduction occurs when 5*‰* dispersed reinforcement is used in mixes containing 7% binder. This effect reverses as the amount of binder is reduced. At lower cement content in the specimens, further strengthening is noticed. Ayeldeen and Kitazume obtained an increase of 240% in unconfined compressive strength with 5*‰* dispersed reinforcement. In this paper, the results obtained for the same reinforcement content increased by 233.65% with mixtures with 3% binder content. Additionally, when the reinforcement content is increased to 25*‰*, it may lead to an increase in unconfined compressive strength in the first days of curing. However, the increase in strength on later days will be restrained by the fibers used [31]. For Bangkok, clays stabilized without reinforcement content according to Lorenzo and Bergado, and the secant modulus ($E_{50}$) can be taken as 115$\;\cdot R_{c}\;$–150 $\;\cdot R_{c}$. In the case of dispersed reinforcement, according to Ayeldeen, the secant modulus ($E_{50}$) can be taken as 95$\;\cdot R_{c}$. In this case, the amount of reinforcement does not affect the results. Comparing the values proposed by other researchers with our own research, common features can be seen. The upper limit of accepted values of modulus ($E_{50}$) for soils without dispersed reinforcement content is 150$\;\cdot R_{c}$. From our own research, this value was 164.64$\cdot R_{c}$ before interpretation. After its interpretation, this value increased to 324.785$\;\cdot \;R_{c}$. The effect of eliminating the influence of the first stage of the test can be seen, which is the adaptation of the machine piston to the sample. The authors proposed a different approach to determine the secant modulus ($E_{50}$) when using dispersed reinforcement. Thus, in the case of using 2.5*‰* reinforcement, the value of modulus ($E_{50}$) can be taken as 137.386$\;\cdot R_{c}$ and 115.329$\;\cdot R_{c}$ for 5*‰*. With the interpretation made, these values increased to 272.177$\;\cdot R_{c}$ and 200.018$\;\cdot R_{c}$, respectively. 

The presented study confirmed the influence of dispersed reinforcement on the modification of physical properties and improved mechanical properties of stabilized soils. The study was conducted for clSa soil stabilized with 3%, 5%, and 7% blast furnace cement (BFC) with the addition of dispersed reinforcement in the form of polypropylene (PP) fibers with a length of 12 mm. The amount of reinforcement used in the mixes was 0*‰*, 2.5*‰,* and 5*‰*.

1. The use of dispersed reinforcement reduces the mix’s maximum dry density (MDD) and increases the optimum moisture content. This effect increases as more fiber is used.
2. The dispersed reinforcement has a more significant effect on the ultimate compressive strength with a lower binder content (3% BFC), where more than a doubling of the unconfined compressive strength can be achieved. The paper does not specify a lower limit for the amount of BFC at which the effect of dispersed reinforcement is still noticeable.
3. In soils stabilized with 3% blast furnace cement (BFC), the use of dispersed reinforcement increases its strength to 221.54% in the first 7 days and to 233.65% after 28 days. As the cement content increases, the effect of polypropylene fibers is reduced. In mixes containing 7% blast furnace cement (BFC), the application of 5% dispersed reinforcement causes weakening of the mixture.
4. The use of polypropylene (PP) fibers as dispersed reinforcement increase the elastic moduli ($E_{s}$, $E_{50}$, $E_{t}$) of soils stabilized by 3% blast furnace cement (BFC), compared to mixtures without dispersed reinforcement. The value of moduli increased up to 230.11%. As the amount of binder increases, the strengthening effect is weaker. The use of distributed reinforcement in soils stabilized by 7% blast furnace cement (BFC) results in elastic reduction up to 39.9%
5. The elimination of the first phase of the test, which is the adaptation of the piston to the specimen, through the interpretation of the results, leads to increased values of the secant moduli ($E_{s}$ and $E_{50}$). The $E_{50}$ values are similar to the tangent modulus $E_{50}$ after interpretation. The unconfined compressive strength is not affected by the interpretation.
6. Despite the increase in the tested specimens’ elastic, with the same binder content, due to the use of dispersed reinforcement, it is with the relationship of elastic modulus with unconfined compression strength that the material weakens. In the case of reinforcement with and without reinforcement for the same unconfined compression strengths, not the cement content, a lower modulus will be obtained in mix with dispersed reinforcement.
7. By adding to the soil and binder mixture, there is a dispersed reinforcement in an optimum amount. It is possible to reduce the weight of cement used in the mixture while maintaining similar values of unconfined compression strength. Considering the high CO2 emission during cement production, it is important to constantly search for methods that can reduce the consumption of this material.

## Figures and Tables

**Table 1:** Properties of the soil.

Physical Properties of Soil	Value	Units
d_10_	0.0025	(mm)
d_30_	0.0390	(mm)
d_60_	0.1300	(mm)
C_C_	4.60	(-)
C_U_	52	(-)
PI	10.41	(-)
LI	0.31	(-)
W_L_	18	(%)
W_p_	7.59	(%)
W	10.8	(%)
pH	8.95	(-)
MDD	2.08	(g × cm^−3^)
OMC	8.64	(%)
Cohesion	7	(kPa)
Internal friction angle	34.5	(°)

**Table 2:** Properties of blast furnace cement.

Properties of Binder	Value	Units
Binder type	blast furnace cement	(-)
Designation	CEM III/A 32,5N-LH/HSR/NA	(-)
Required compressive strength after 7 days	≥16.0	(MPa)
Required compressive strength after 28 days	≥32.5 ≤52.5	(MPa) (MPa)
Hydration heat	≤270	(J/g)

**Table 3:** Properties of polypropylene fibers.

Properties of Fibers	Value	Units
Polymeric type	Polypropylene	(-)
Single fiber length	12	(mm)
Diameter of a single fiber	32	(µm)
Tensile strength	300 ÷ 400	(MPa)

**Table 4:** Overview of the mixtures.

Mixture Name	Binder (%)	Fibers (‰)	Day (-)	Mixture Name	Binder (%)	Fibers (‰)	Day (-)
C102	3	0	7	C104	3	0	28
C112	3	2.5	7	C114	3	2.5	28
C122	3	5	7	C124	3	5	28
C202	5	0	7	C204	5	0	28
C212	5	2.5	7	C214	5	2.5	28
C222	5	5	7	C224	5	5	28
C302	7	0	7	C304	7	0	28
C312	7	2.5	7	C314	7	2.5	28
C322	7	5	7	C324	7	5	28

**Table 5:** Basic information about the instrument and measurement.

Details	Value	Units
Universal Testing System	Instron	(-)
Model	5982	(-)
Force Measurement Accuracy	±0.5	(%)
Displacement Measurement Accuracy	±0.01	(mm)
Testing Speed Accuracy:	±0.1	(%)
Data Acquisition Rate at the PC	2.5	(kHz)
Load speed	16	(kN/min)
Test control program	Bluehill	(-)

**Table 6:** Summary of unconfined compression strength results.

Mixture Name	R_c_ (MPa)	R_c_ Category	Change (%)	Mixture Name	R_c_ (MPa)	R_c_ Category	Change (%)
C102	0.65	C_0.4/0.5_	100.00	C104	1.04	C_0.8/1.0_	100.00
C112	1.21	C_0.8/1.0_	186.15	C114	2.28	C_1.5/2.0_	219.23
C122	1.44	C_0.8/1.0_	221.54	C124	2.43	C_1.5/2.0_	233.65
C202	1.45	C_0.8/1.0_	100.00	C204	2.92	C_2.0/2.5_	100.00
C212	1.99	C_1.2/1.5_	137.24	C214	3.65	C_2.3/3.0_	125.00
C222	2.01	C_1.5/2.0_	138.62	C224	3.63	C_2.3/3.0_	124.32
C302	2.52	C_2.0/2.5_	100.00	C304	4.95	C_3.0/4.0_	100.00
C312	2.94	C_2.0/2.5_	116.67	C314	5.25	C_4.0/5.0_	106.06
C322	2.69	C_2.0/2.5_	106.75	C324	4.73	C_3.0/4.0_	95.56

**Table 7:** Effect of interpretation on modulus values $E_{s}$.

Mixture Name	$E_{s}$ After (MPa)	$E_{s}$ Before (MPa)	Change (%)	Mixture Name	$E_{s}$ After (MPa)	$E_{s}$ Before (MPa)	Change (%)
C102	55.45	62.97	113.56	C104	83.72	111.31	132.96
C112	81.55	113.42	139.08	C114	136.12	164.83	121.09
C122	53.07	61.83	116.51	C124	127.1	144.55	113.73
C202	68.59	84.26	122.85	C204	155.65	179.53	115.34
C212	96.16	112.76	117.26	C214	159.43	197.53	123.89
C222	68.33	77.96	114.09	C224	148.44	185.03	124.65
C302	188.57	248.01	131.52	C304	346.49	478.96	138.23
C312	159.02	199.71	125.59	C314	342.72	440.77	128.61
C322	86.35	96.48	111.73	C324	214.13	253.00	118.15

**Table 8:** Summary of unconfined compression strength results.

Mixture Name	$E_{50}$ After (MPa)	$E_{50}$ Before (MPa)	Change (%)	Mixture Name	$E_{50}$ After (MPa)	$E_{50}$ Before (MPa)	Change (%)
C102	68.72	91.07	132.52	C104	86.75	163.43	188.39
C112	86.20	209.56	243.11	C114	158.44	268.95	169.75
C122	79.95	128.74	161.03	C124	194.70	303.91	156.09
C202	85.87	145.64	169.91	C204	281.81	432.92	153.72
C212	125.17	197.12	157.48	C214	208.81	427.07	204.53
C222	105.11	162.61	154.70	C224	186.96	370.33	198.08
C302	214.88	469.55	218.52	C304	386.12	832.32	215.56
C312	187.97	365.64	194.52	C314	403.70	824.36	204.20
C322	157.06	268.05	170.67	C324	267.56	463.79	173.34

**Table 9:** Parameter A for determining the prediction of modulus values for stabilized soils.

Fibers Content of the Stabilized Soil [‰]	E (Type) (MPa)	*A* (-)	r^2^ (-)
Results without interpretation of measurement data			
0.0	$E_{s}$	66.701	0.915
0.0	$E_{50}$	164.640	0.707
0.0	$E_{t}$	164.116	0.882
2.5	$E_{s}$	57.218	0.835
2.5	$E_{50}$	137.386	0.844
2.5	$E_{t}$	139.256	0.769
5.0	$E_{s}$	42.120	0.858
5.0	$E_{50}$	115.329	0.696
5.0	$E_{t}$	100.065	0.705
**Results with interpretation of measurement data**			
0.0	$E_{s}$	88.257	0.880
0.0	$E_{50}$	324.785	0.875
0.0	$E_{t}$	164.116	0.882
2.5	$E_{s}$	72.324	0.807
2.5	$E_{50}$	272.177	0.809
2.5	$E_{t}$	139.256	0.769
5.0	$E_{s}$	49.734	0.883
5.0	$E_{50}$	200.018	0.710
5.0	$E_{t}$	100.065	0.705

## Data Availability

The data presented in this paper are available upon request from the authors. These data are not publicly available due to ongoing follow-up work.
